# Supplementary material for: Direct chiroptical correlation of dissymmetric crystal morphologies
Source: Nat Commun. 2025 Sep 26;16:8441. doi: 10.1038/s41467-025-62889-2 (PMC12474863; doi:10.1038/s41467-025-62889-2)
Supplement: Supplementary file 1 — Supplementary Information [file 41467_2025_62889_MOESM1_ESM.pdf]

Supplementary Information

Direct Chiroptical Correlation of Dissymmetric Crystal Morphologies

Qiang Wen[1], Melissa Tan[2], Ofir Eisenberg[1], Hadar Nasi[1],
Vlad Brumfeld[3], Akash Tiwari[2], Hai-Mu Ye[2], Bart Kahr[2,*], Linda J. W. Shimon[3,*],
Michal Lahav[1,*] and Milko E. van der Boom[1,*]

[1]Department of Molecular Chemistry and Materials Science, Weizmann Institute of Science, Rehovot 7610001, Israel.

[2]Department of Chemistry and Molecular Design Institute, New York University, 100 Washington Square East, New York, New York 10003, United States.

[3]Department of Chemical Research Support, Weizmann Institute of Science, Rehovot 7610001, Israel.

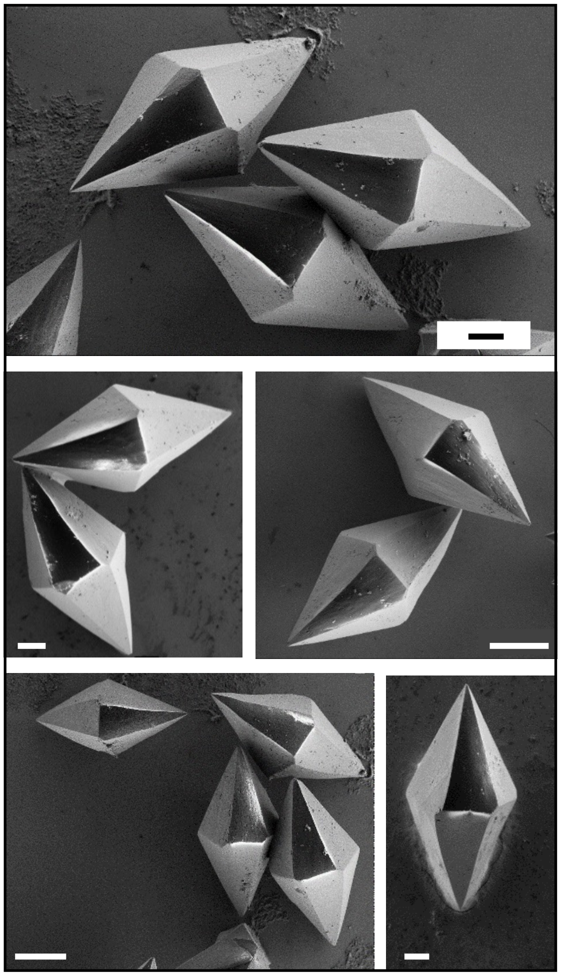

Fig. S1 Scanning electron microscopy (SEM) images of chiral hexagonal trapezohedral crystals obtained using solvothermal conditions. Scale bar = 10 μm.

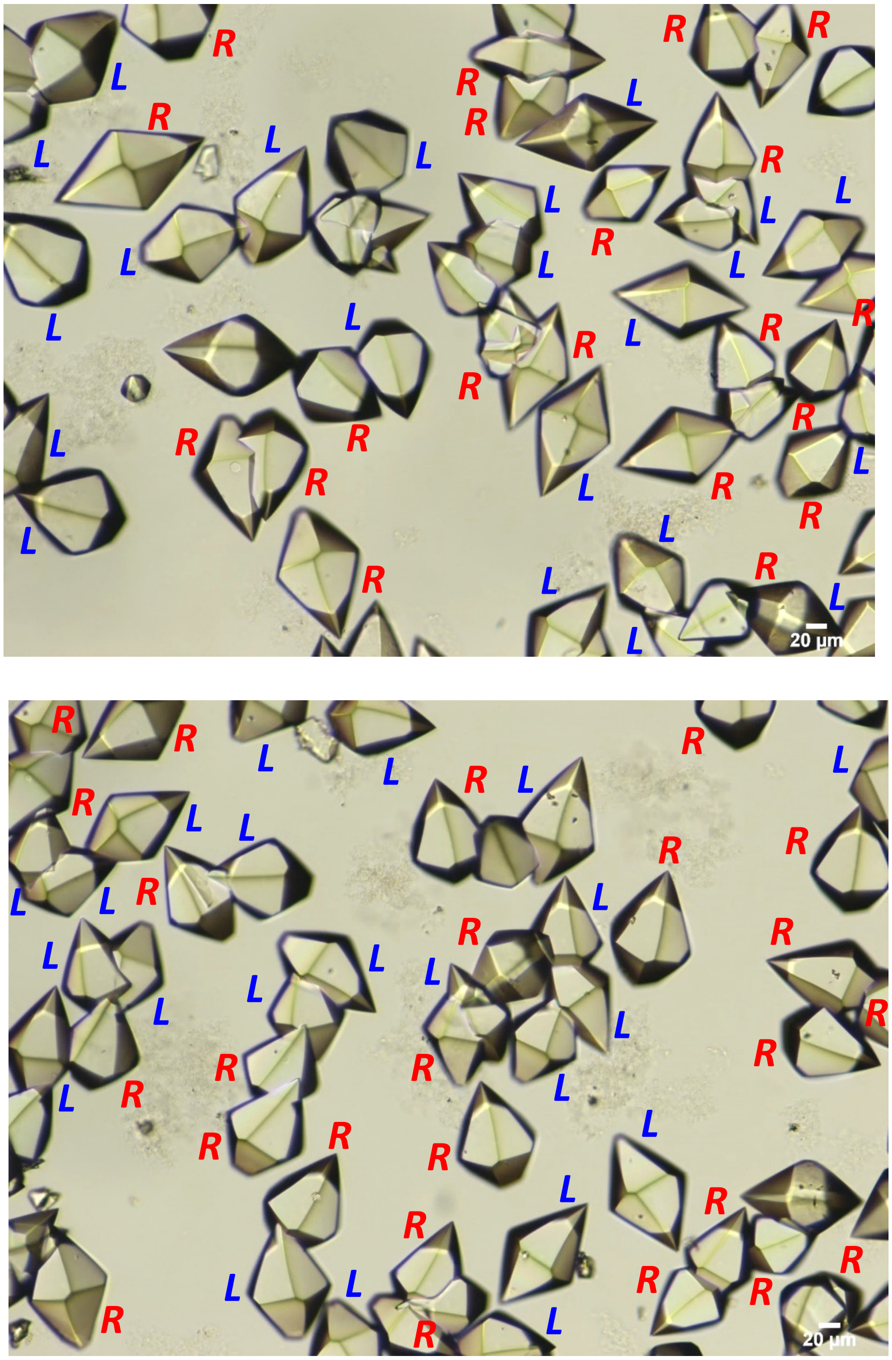

Fig. S2. Optical micrographs of chiral hexagonal trapezohedral crystals with R = right-handiness, L = left-handiness. The crystals were prepared using solvothermal conditions.

Table S1. Statistical analysis of the chirality from five different batches. The crystals were prepared using solvothermal conditions.

Batch	L-crystals	R-crystals	L/R (%/%)
1	422	404	52/48
2	476	516	48/52
3	476	456	51/49
4	432	427	50/50
5	523	480	52/48

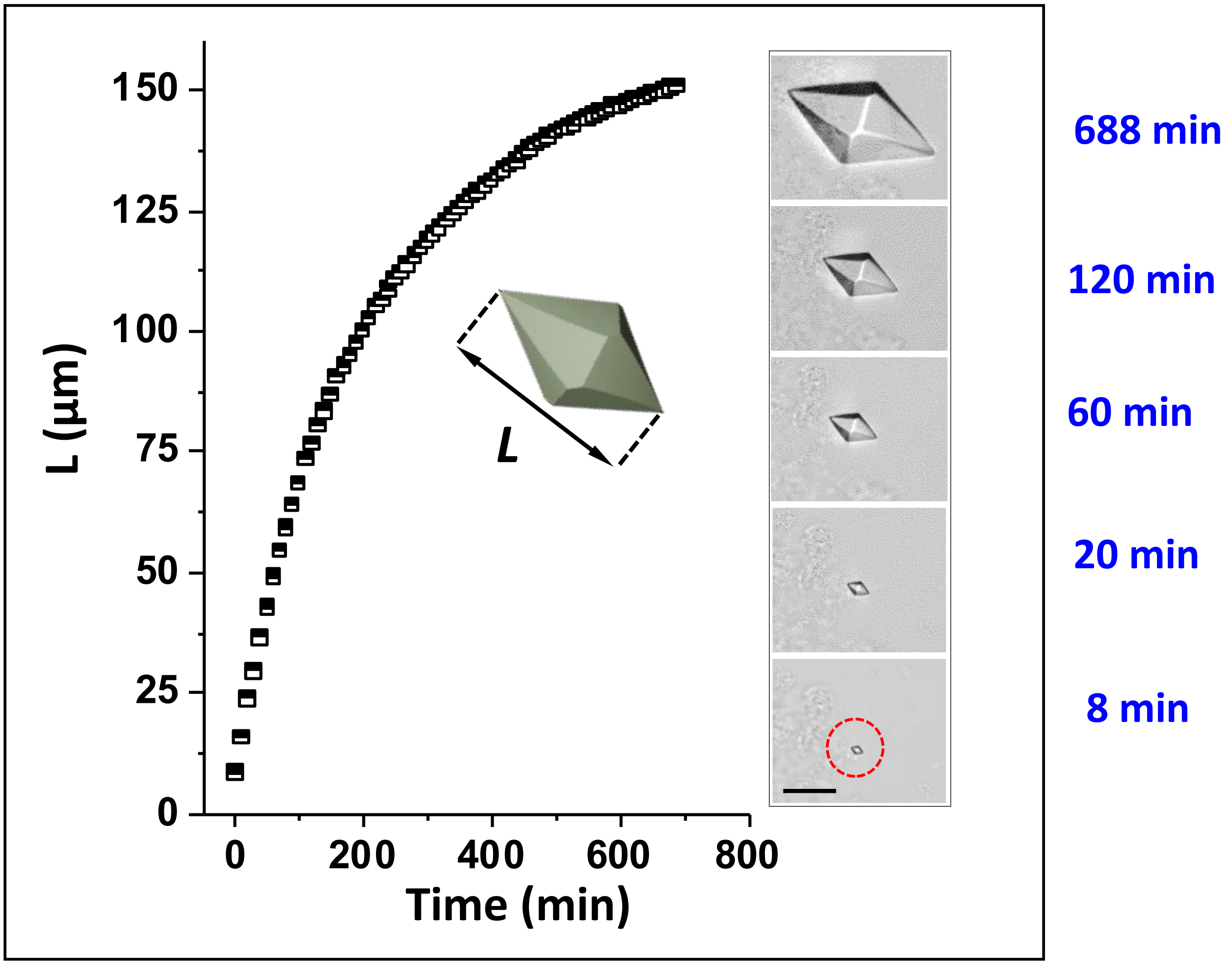

Fig. S3. *In situ* monitoring of the crystal growth at room temperature by inverted optical microscopy (Nikon Eclipse Ti2-E). The supersaturated solution was prepared using solvothermal conditions. The images were automatically taken every minute. Scale bar = 50 μm.

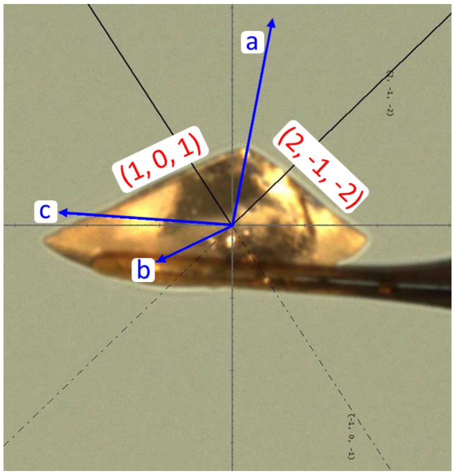

Fig. S4 Example of morphology planes analysis (*hkl*) of QW-MOF using solvothermal conditions. The optical microscopy image show a crystal mounted on a loop. The individual planes (1, 0, 1) and (2, $\bar{1}$, $\bar{2}$) were indexed by CrysAlisPro. The crystallographic axes are marked in blue. Note that since the symmetry of the crystal structure is 622, the indices could be assigned with the opposite polarity, i.e. (1,0, $\bar{1}$), and (2, $\bar{1}$, 2). The indices are in accord with the models in the manuscript.

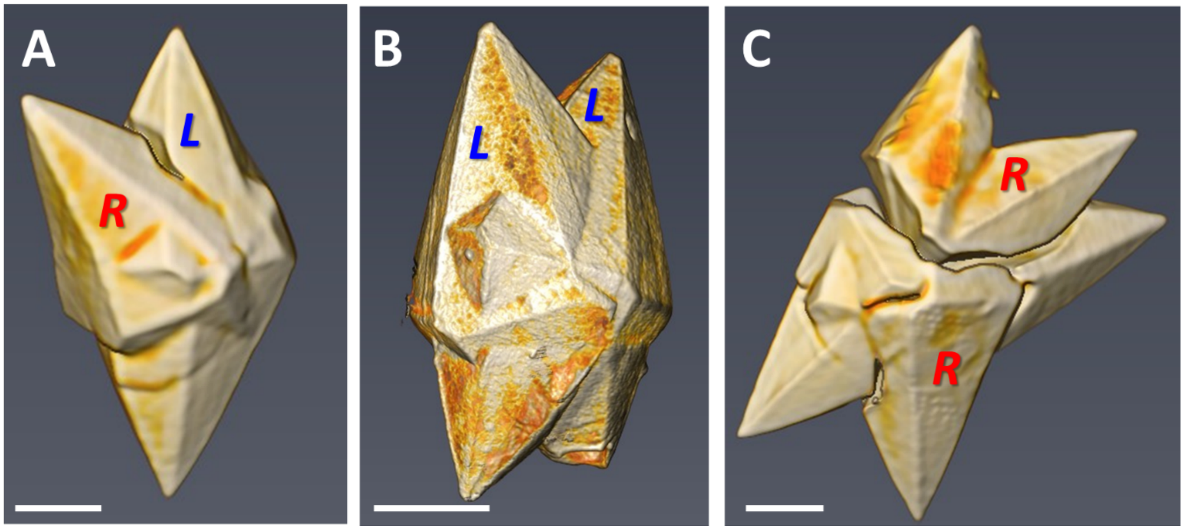

Fig. S5. X-ray micro-computed tomography (microCT) of interpenetrated twinned crystals. Scale bar = 25 μm. The crystals were prepared using solvothermal conditions.

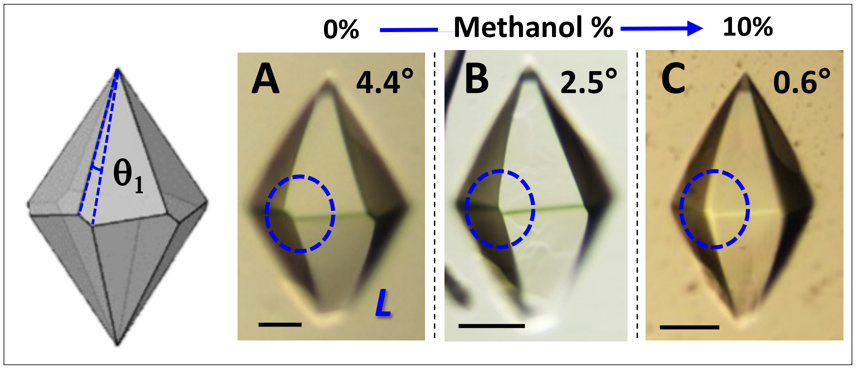

Fig. S6. Crystals prepared by layering using different amounts of methanol. The degree of the off-set angle decreases with increasing the amount of methanol (%): (a) 0%: 4.4° (±0.3). (b) 5%: 2.5° (±0.5), (c) 10%: 0.6° (±0.3), (scale bar = 25 μm). Optical images of the *R*-twisted" crystals are shown in **Fig. 2E-G**. Solvothermal conditions without methanol afforded crystals having an off-set angle of 5.8° (±0.2).

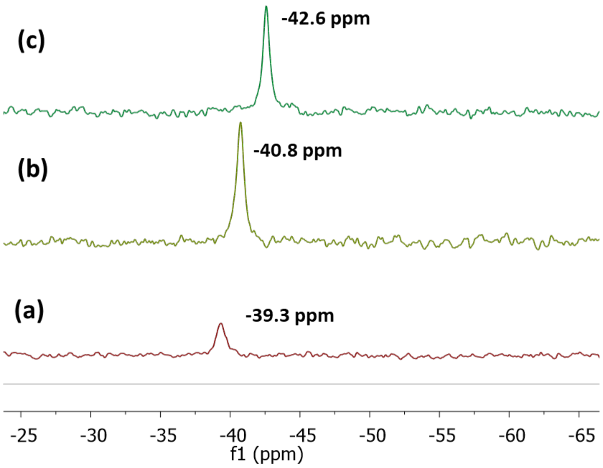

Fig. S7. ^{113}Cd NMR of $Cd(OAc)_2$ in (a) DMF, (b) DMF (5% CH_3OH), (c) DMF (10% CH_3OH).

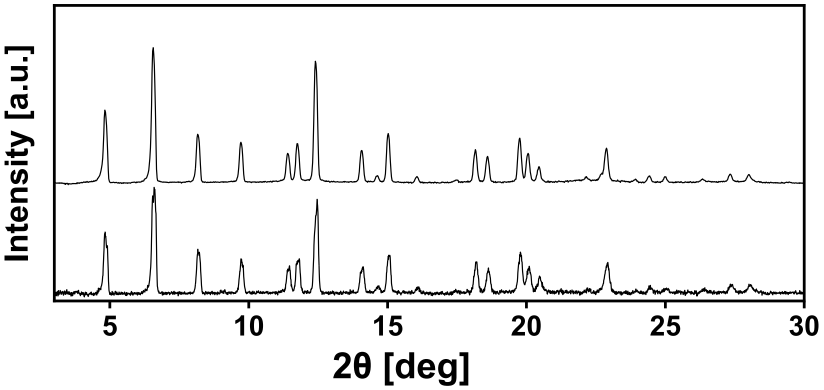

	Unit cell dimensions		
	a=b	c	Angles [0]
**v483**	14.9799 (2)	18.3087 (3)	$\alpha = \beta = 90$<br>$\gamma = 120$
**Twisted PXRD**	15.460 (0.001)	18.139 (0.002)	$\alpha = \beta = 90.0$<br>$\gamma = 120.0$
**Non Twisted PXRD**	15.495 (0.004)	18.169 (0.0)	$\alpha = \beta = 90.0$<br>$\gamma = 120.0$

Fig. S8. Powder X-ray diffraction (PXRD) data of QW-MOF. Non-twisted (top) and twisted-crystals (bottom). Both samples were prepared at room temperature using layering. The isohedral sample was obtained by using 10% methanol. The data was collected by measuring the samples in a rotating capillary along with the reaction solution. The data indicate a *P*622 space group.

Optical activity of the ethylenediammonium sulfate (EDS) model crystals

Ethylenediammonium sulfate (EDS) was first crystallized in the nineteenth century. Crystals of EDS grow as transparent, tetragonal bipyramids with good basal cleavage and high rotatory power. This system has been well studied.[1-4] Full dispersion relations for the permittivity and gyration of EDS were recently determined by generalized ellipsometry.[5] Dextro- and levo-rotatory enantiomorphs were easily distinguished by analyzing basal slabs with a polarizing microscope. Starting with crossed polarizers, if extinction arises with a clockwise rotation of the analyzer, the structure is dextrorotatory ($P4_32_12$). Conversely, if a counterclockwise rotation generates extinction, the crystal is levorotatory ($P4_12_12$).[6]

Mueller Matrix Polarimetry

The optical properties of a sample can also be determined analytically by inversion.[6,7] This approach recasts an experimental Mueller matrix in the form of a Jones matrix $\mathbf{J} = \begin{pmatrix} J_{11} & J_{12} \\ J_{21} & J_{22} \end{pmatrix}$ expressed as:

$$J_{11} = \sqrt{\frac{M_{11} + M_{12} + M_{21} + M_{22}}{2}}$$

$$J_{12} = \frac{\left(M_{13} + M_{23} - i(M_{14} + M_{24})\right)}{2J_{11}}$$

$$J_{21} = \frac{\left(M_{31} + M_{32} + i(M_{41} + M_{42})\right)}{2J_{11}}$$

$$J_{22} = \frac{\left(M_{33} + M_{44} + i(M_{43} - M_{34})\right)}{2J_{11}}.$$

$LR, LR', and\ CR$ are subsequently determined as:

$$\kappa = -Im\big(2iln(K)\big)$$

$$LR = \big(i\Omega(J_{11} - J_{22})\big)$$

$$LR' = \big(i\Omega(J_{12} + J_{21})\big)$$

$$CR = \big(i\Omega(J_{12} - J_{21})\big)$$

$$K = 1/\sqrt{\det(\mathbf{J})}$$

$$\mathcal{T} = 2acos(K(J_{11} + J_{22})/2)$$

$$\Omega = \frac{K(\mathcal{T} + 2\pi n)}{2\sin\left(\frac{\mathcal{T}}{2}\right)}$$

where quantities LR $(LR'), CR$, are respectively linear and circular retardances, and κ is mean extinction. The primed quantities are referred to a coordinate system that is diagonal to that for the quantities without the primes. All have units in radians.

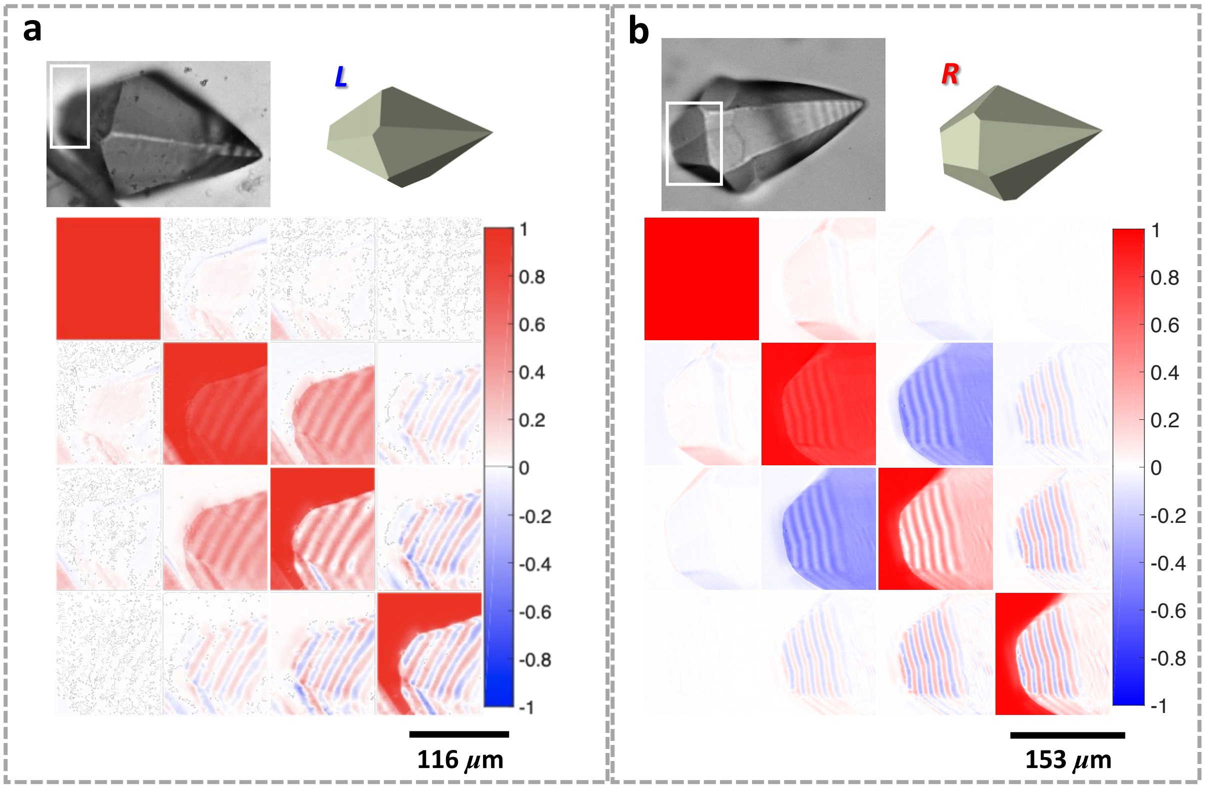

Fig. S9 Normalized transmission Mueller matrix (M) corresponding to (a) a left-handiness crystal, and (b) a right-handiness crystal (λ = 550 nm).

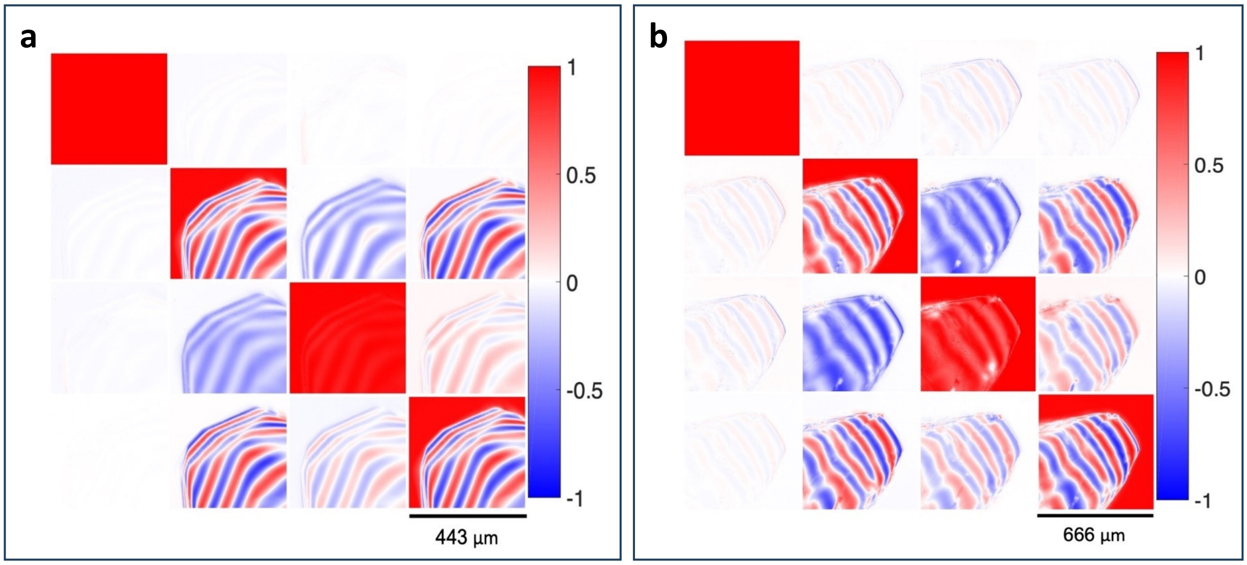

Fig. S10 Normalized transmission Mueller matrix (M) corresponding to (a) $P4_12_12$ and (b) $P4_32_12$ enantiomorphs of ethylenediammonium sulfate (EDS; λ = 480 nm).[4]

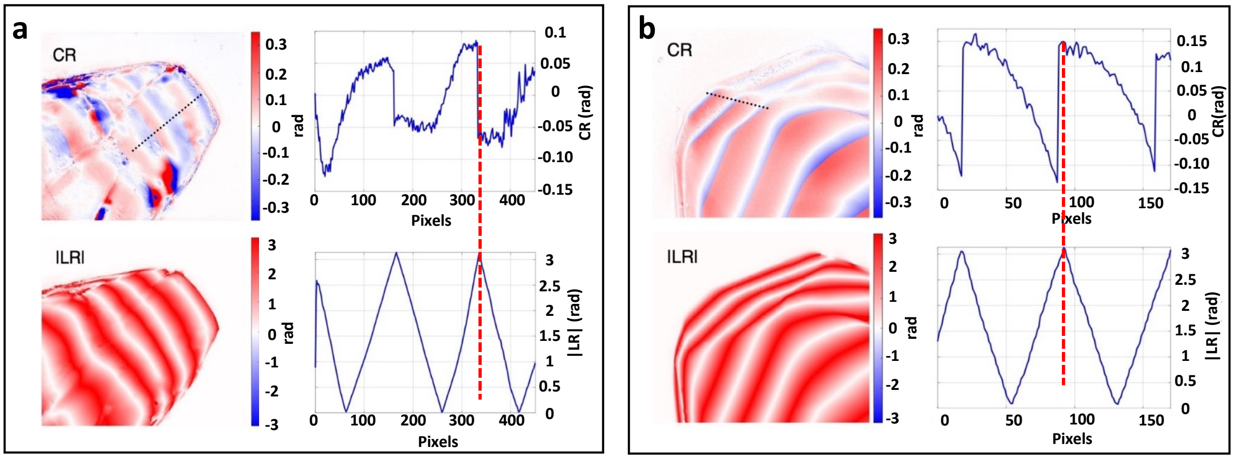

Fig. S11 Interdependence of bianisotropies CR and |LR| determined from normalized transmission Mueller matrix measurement on (a) $P4_12_12$ and (b) $P4_32_12$ enantiomorphs of ethylenediammonium sulfate (EDS; λ = 480 nm). The 1D plots are drawn along the path shown in the CR micrograph indicated with a black dash line.[4]

Table S2. The crystal data and the structural refinement of QW-MOF.

Crystal	V483
CCDC	2368214
Crystal description	hexagonal trapezohedral
Source	Rigaku Synergy-R
Empirical formula	$C_{105}H_{90}CdN_6$, 2[$C_2H_3O_2$], 2H_2O
Formula weight (g/mol)	1702.34
Temperature (K)	100
Wavelength (Å)	1.54184
Crystal system	hexagonal
Space group	*P*622
a (Å)	14.9799(2)
b (Å)	14.9799(2)
c (Å)	18.3087(3)
α, β, γ°	90, 90, 120
Volume (Å^3)	3558.00(11)
Z	2
Density calculated (Mg/m^3)	1.455
Absorption coefficient (mm^{-1})	2.925
F(000)	1784
Theta range for data collection (°)	3.407 to 47.214
Reflection collected (Unique)	77579(1102)
R_{int}	0.021
Completeness %	99.8
Data/restraints/parameters	1102/184/111
Goodness-of-fit; F^2	3.034
Final R [I>2σ(I)]	R1 = 0.2450<br>wR2 = 0.5525
R (all data)	R1= 0.2615<br>wR2 = 0.5944
Largest diff. peak and hole (e$\cdot$ Å^{-3})	2.789 and<br>-0.799
Flack parameter	0.28(11)

References

1 Baldwin, W. Rotatory dispersion in the amine series. IV. The optical activity of diamines. *Proc. Roy. Soc. London. Ser. A. Math. Phys. Sci.* **167**, 539-554 (1938).

2 Koby, L., Ningappa, J., Dakessian, M. & Cuccia, L. Chiral cystallization of ethylenediamine sulfate. *J. Chem. Ed.* **82**, (2005).

3 Martin, A. T. Optical activity anisotropy in solids, Doctoral dissertation, New York University, 2018.

4 Tan, S. C. M. The chiroptics of imperfect crystals, Doctoral dissertation, New York University (2020).

5 Nichols, S., Martin, A., Choi, J. & Kahr, B. Gyration and permittivity of ethylenediammonium sulfate crystals. *Chirality* **28**, 460-465 (2016).

6 Matsumoto, A., Ide, T., Kaimori, Y., Fujiwara, S. & Soai, K. Asymmetric autocatalysis triggered by chiral crystal of achiral ethylenediamine sulfate. *Chem. Lett.* **44**, 688-690, (2015).

7 Arteaga, O. & Canillas, A. Analytic inversion of the Mueller–Jones polarization matrices for homogeneous media, *Opt. Lett.* 35, 559-561 (2010).

8 Nichols, S. Coherence in polarimetry, Doctoral dissertation, New York University, (2018).
