# Supplementary material for: Direct chiroptical correlation of dissymmetric crystal morphologies
Source: Nat Commun. 2025 Sep 26;16:8441. doi: 10.1038/s41467-025-62889-2 (PMC12474863; doi:10.1038/s41467-025-62889-2)
Supplement: Supplementary file 3 — Description of Additional Supplementary Files [file 41467_2025_62889_MOESM3_ESM.pdf]

Description of Additional Supplementary Files

File name: Supplementary Movie 1

Description: microCT volume rendering of a crystal.

File name: Supplementary Movie 2

Description: microCT volume rendering of a crystal.
